# The marine phosphorus cycle driven by an unlikely microbe

**DOI:** 10.1038/s41467-025-64456-1

**Published:** 2025-11-07

**Authors:** Senjie Lin, Alexander Francoeur

**Affiliations:** Department of Marine Sciences, University of Connecticut, Groton, CT USA

**Keywords:** Element cycles, Environmental microbiology, Element cycles

## Abstract

Phosphorus availability is vital to ocean ecosystem functioning. Analyses of global seawater samples combined with laboratory experiments reveal the *Alteromonas* bacteria as a surprising regulator of the marine phosphorus cycling.

Phosphorus forms key components of nucleic acids, proteins, and cell membranes and is vital for life. Yet, the primary form of bioavailable phosphorus, dissolved inorganic phosphorus (Pi), is often limited in the ocean due to slow geochemical cycling and lack of biogenic sources^1,2^. In general, phosphorus limitation influences the metabolisms of bacteria^3^ and phytoplankton^4,5^, which impacts the cycling of carbon and other important nutrients. To overcome phosphorus scarcity, marine microbes have evolved alkaline phosphatase (APase) enzymes that convert dissolved organic phosphorus into bioavailable phosphorus that can be consumed by organisms. Despite the central role of APases in the marine phosphorus cycle, much about their functionality and expression remain unknown. To address this gap, Saavedra and colleagues^6^ perform an extensive global assessment of microbial sequences and reveal the bacterial group *Alteromonas* as the dominant contributor to microbial APase activity in the global ocean. Following clues from the sequencing data, the authors take to the laboratory and demonstrate that most APase types in an *Alteromonas* species are constantly expressed, challenging the widely accepted notion that APase is induced by phosphorus deficiency.

## Alkaline phosphatase: one enzyme, multiple layers of complexity

Alkaline phosphatase is a critical enzyme in the marine phosphorus cycle and is generally regarded as a specific indicator of phosphorus stress. However, despite extensive study, the complexity of this enzyme leaves open questions related to its functional diversity, environmental distribution, and the microbes responsible for generating it. Many APase forms have been described, with various subcellular localizations as well as prevalence in surface and deep ocean waters^7–10^. These forms differ in their building block requirements (metal cofactors) and substrate specificity (the molecules they act on). Some APase forms are even extracellular (cell-free), meaning that their expression benefits neighboring microbes by increasing bioavailable phosphorus. These extracellular APases are especially beneficial in areas of low nutrients, such as subtropical gyres and the Mediterranean Sea.

In the deep ocean, where phosphorus is not limited, researchers have observed a high prevalence of extracellular forms of APase^11^. This unexpected phenomenon, known as the “APase paradox”, has garnered recent attention, with competing explanations^12^. While we know that APases from different microbes and phytoplankton conceivably function in a highly adaptive and complementary manner, it remains unclear if the APase paradox is connected to the extracellular APase expression of a specific microbial group.

## The latest twist: *Alteromonas* dominates microbial APase

**Fig. 1:**
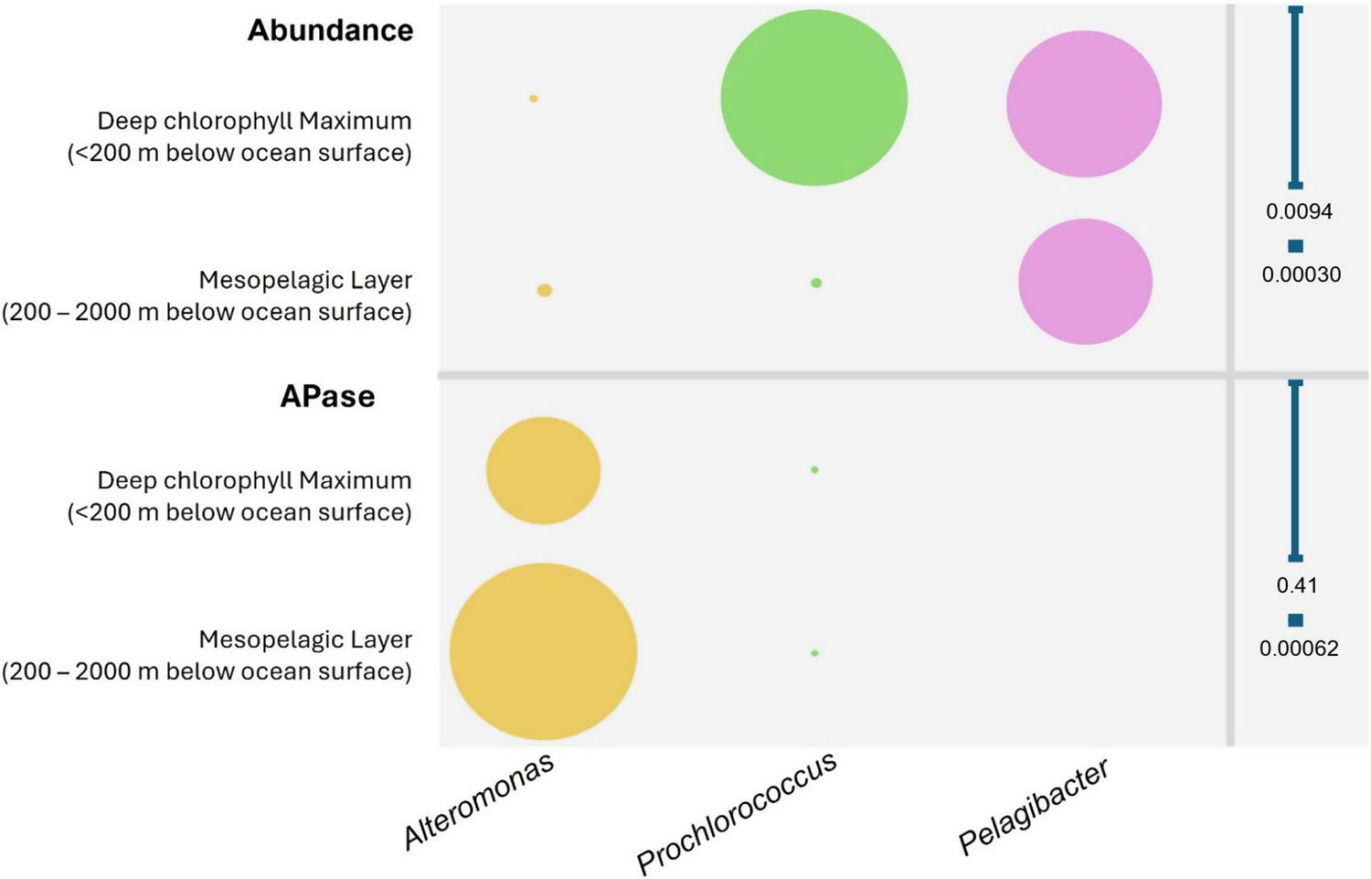
Contrasting gene abundance (top) and APase transcript abundance (bottom) in *Alteromonas*, *Prochlorococcus*, and *Pelagibacter.* *Alteromonas* punches above its weight in the marine phosphorus cycle. Though outnumbered in abundance in the ocean, the bacterial group *Alteromonas* dominates APase transcripts and proteins in both the surface ocean (Deep Chlorophyll Maximum) and deeper layers (Mesopelagic Layer)^1^. Circle size represents relative abundance (fraction of community total 16S reads; top panel) or APase activity (fraction of community total PhoA transcripts; bottom panel), with scales shown with vertical lines on the right.

In their quest to better understand APase distribution and activity, Saavedra and colleagues^6^ discover that the bacterial group *Alteromonas* dominates microbial APase generation in the global ocean. Though *Alteromonas* is a widely distributed heterotrophic bacterium, found from surface oceans to deep-sea sediments, its relative abundance is dwarfed by that of the photoautotroph *Prochlorococcus*, which uses light for energy, and heterotroph *Peliagibacter*, which consumes organic compounds for energy (top of Fig. 1). *Alteromonas* is hundreds of times less abundant than *Prochlorococcus* at the deep chlorophyll maximum (DCM) layer (<~200 m below the surface) and *Peliagibacter* at the mesopelagic (MES) layer (200–2000 m below the surface). The dominance of *Alteromonas* APase transcripts and proteins in the deep ocean is counterintuitive because of their lower abundance. This suggests that despite their lower abundance, this group has much higher APase synthesis rate, stability, and activity compared to other microbial groups. Therefore, *Alteromonas* is the hidden gem in marine phosphorus cycling and other related biogeochemical cycles.

The authors also shed light on *Alteromonas*’ role as the missing link in the APase paradox in the deep ocean. Using a combination of molecular analyses and rate measurements, they find that even under conditions where bioavailable phosphorus is not limited, APase is expressed. This suggests that expression of APase by *Alteromonas* is decoupled from P-limitation responses and is constitutive, always present and continuously synthesized. Likely, APase expression in this group of bacteria is regulated by factors other than phosphate availability and serves to provide organic carbon breakdown products for these bacteria^12^, while releasing phosphate to benefit other microbes in deep water or, through upwelling, to phytoplankton in the surface. Therefore, the role of *Alteromonas* in marine P cycling and other related biogeochemical cycles is likely more prominent than currently believed.

## Unresolved mysteries of a classic enzyme

**Fig. 2:**
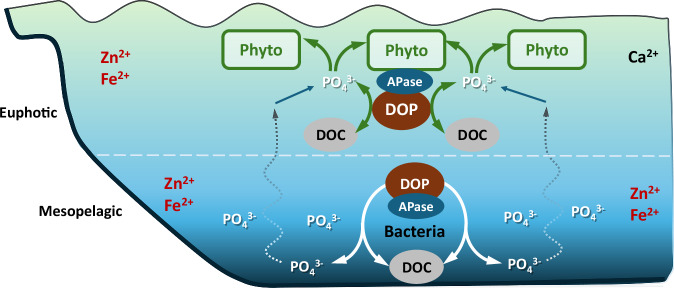
Schematic of the different roles of alkaline phosphatase (APase). Upwelling currents in the ocean bring bioavailable phosphate generated by mesopelagic bacteria to the euphotic zone where it can be used by phytoplankton (Phyto). These phytoplankton also express APases to obtain phosphorus from dissolved organic phosphorus (DOP), releasing dissolved organic carbon (DOC). Future research should focus on potential differences between euphotic zone phytoplankton and mesopelagic microbes as it relates to phosphorus and carbon biogeochemical cycling, and the use of metal cofactors.

Saavedra and colleagues, along with recent literature, emphasize questions around the adaptive evolution and ecological roles of APase. Despite the emerging understanding that *Alteromonas* bacteria play a disproportionate role in the regulation of the marine phosphorus cycle, we are yet to understand whether APases produced by mesopelagic microbes and euphotic-zone phytoplankton serve different functions for these organisms (Fig. 2). Such potential differences have implications for the carbon cycle, depending upon the type of phosphorus-containing compound that is being broken down. Additional focused systematic studies of carbon pools, phosphorus availability, APase activity, and taxonomic analyses are needed. Moreover, we do not yet know whether phytoplankton APase activities are dominated by one or a few groups, as we now understand to be the case for bacteria^6^. The dominant phytoplankton groups could be the same as those responsible for harmful algal blooms that spit out environmental toxins in phosphate-limited environments. If so, current biogeochemical and harmful algal bloom models may need to be revised.

Though the work by Saavedra and colleagues represents a snapshot in time, it sets up questions related to future oceanic conditions and APase dynamics. Changes in ocean chemistry and stratification with global warming may affect the distribution, cycling, and transport of phosphorus^13^. Would this change impact APase activity and the groups of organisms responsible for APase synthesis? Also, if different APase types align with trace metal availability in the ocean according to their cofactor requirements^14^ (Fig. 2), would changes in the global ocean impact APase distribution, as well? How would the spatial and temporal distribution of these APases, along with their different substrate specificities as found in this and previous^15^ studies, impact the landscape of phosphorus and carbon biogeochemical cycles? These are all intriguing questions that Saavedra and colleagues spur with their new work.

In sum, while many questions about APase exist, the clever combination of laboratory and sequencing analyses uncovered *Alteromonas* as a surprising powerhouse in phosphorus cycling. This humble bacterium dominates the production of APase in the ocean, proving that key forces in global biogeochemical processes are not always the most abundant.

## References

[1] Paytan, A. & McLaughlin, K. The oceanic phosphorus cycle. *Chem. Rev.* **107**, 563–576 (2007). 17256993 10.1021/cr0503613

[2] Ustick, L. J. et al. Metagenomic analysis reveals global-scale patterns of ocean nutrient limitation. *Science* **372**, 287–291 (2021). 33859034 10.1126/science.abe6301

[3] Lamarche, M. G., Wanner, B. L., Crepin, S. & Harel, J. The phosphate regulon and bacterial virulence: a regulatory network connecting phosphate homeostasis and pathogenesis. *FEMS Microbiol. Rev.* **32**, 461–473 (2008). 18248418 10.1111/j.1574-6976.2008.00101.x

[4] Li, M., Li, L., Shi, X., Lin, L. & Lin, S. Effects of phosphorus deficiency and adnosiine 5’-triphosphate (ATP) on growth and cell cycle of the dinoflagellate *Prorocentrum donghaiense*. *Harmful Algae* **47**, 35–41 (2015).

[5] Brembu, T., Mühlroth, A., Alipanah, L. & Bones, A. M. The effects of phosphorus limitation on carbon metabolism in diatoms. *Philos. Trans. R. Soc. Lond. B Biol. Sci.* **372**, 20160406 (2017). 28717016 10.1098/rstb.2016.0406 PMC5516115

[6] Saavedra, D. E. M. et al. Multifunctionally diverse alkaline phosphatases of *Altermomonas* drive the phosphorus cycle in the ocean. *Nat. Commun*. 10.1038/s41467-025-64455-2 (2025). 10.1038/s41467-025-64455-2 PMC12594817 41203623

[7] Luo, H., Benner, R., Long, R. A. & Hu, J. Subcellular localization of marine bacterial alkaline phosphatases. *Proc. Natl. Acad. Sci. USA* **106**, 21219–21223 (2009). 19926862 10.1073/pnas.0907586106 PMC2795515

[8] Kathuria, S. & Martiny, A. C. Prevalence of a calcium-based alkaline phosphatase associated with the marine cyanobacterium *Prochlorococcus* and other ocean bacteria. *Environ. Microbiol.* **13**, 74–83 (2011). 20649645 10.1111/j.1462-2920.2010.02310.x

[9] Lidbury, I. D. E. A. et al. A widely distributed phosphate-insensitive phosphatase presents a route for rapid organophosphorus remineralization in the biosphere. *Proc. Natl. Acad. Sci. USA* **119**, e2118122119 (2022). 35082153 10.1073/pnas.2118122119 PMC8812569

[10] Torcello-Requena, A. et al. A distinct, high-affinity, alkaline phosphatase facilitates occupation of P-depleted environments by marine picocyanobacterial. *Proc. Natl. Acad. Sci. USA* **121**, e2312892121 (2024). 38713622 10.1073/pnas.2312892121 PMC11098088

[11] Baltar, F. et al. High dissolved extracellular enzymatic activity in the deep central Atlantic ocean. *Aquat. Microb. Ecol.* **58**, 287–302 (2010).

[12] Thomson, B. et al. Resolving the paradox: continuous cell-free alkaline phosphatase activity despite high phosphate concentrations. *Mar. Chem.* **214**, 103671.1–103671.6 (2019).

[13] Slomp, C. P. & Van Cappellen, P. The global marine phosphorus cycle: Sensitivity to oceanic circulation. *Biogeosciences* **4**, 155–171 (2007).

[14] Giovannelli, D. Trace metal availability and the evolution of biogeochemistry. *Nat. Rev. Earth Environ.* **4**, 597–598 (2023).

[15] Zhang, K. et al. Functional differentiation and complementation of alkaline phosphatases and choreography of DOP scavenging in a marine diatom. *Mol. Ecol.* **31**, 3389–3399 (2022). 35445467 10.1111/mec.16475

